# Sulbactam–durlobactam and cefiderocol combination treatment of *Burkholderia cenocepacia*-associated Fitz–Hugh–Curtis syndrome

**DOI:** 10.1128/aac.01542-25

**Published:** 2026-04-30

**Authors:** Bhavita Gaglani, Tessa M. Andermann, Claire Wate, Jorge Oldan, Chirag S. Desai, Steven H. Marshall, Robert A. Bonomo, Ray Coakley, David van Duin, Anne Lachiewicz

**Affiliations:** 1 Department of Medicine and Anesthesiology, Wake Forest School of Medicine 12279 , Winston Salem, North Carolina, USA; 2 Department of Medicine, University of North Carolina School of Medicine 214908, Chapel Hill, North Carolina, USA; 3 Institute of Global Health & Infectious Diseases, University of North Carolina 622440, Chapel Hill, North Carolina, USA; 4 Department of Radiology, Molecular Imaging and Therapeutics, University of North Carolina 2331, Chapel Hill, North Carolina, USA; 5 Division of Abdominal Transplant, Department of Surgery, University of North Carolina 214495, Chapel Hill, North Carolina, USA; 6 Research Service, Louis Stokes Cleveland Department of Veterans Affairs Medical Center 20083, Cleveland, Ohio, USA; 7 Department of Medicine, Case Western Reserve University 220786, Cleveland, Ohio, USA; 8 Department of Pharmacology, Case Western Reserve University 2546, Cleveland, Ohio, USA; 9 Department of Biochemistry, Case Western Reserve University 2546, Cleveland, Ohio, USA; 10 Department of Proteomics, Case Western Reserve University 2546, Cleveland, Ohio, USA; 11 Department of Bioinformatics, Case Western Reserve University 2546, Cleveland, Ohio, USA; 12 Department of Molecular Biology and Microbiology, Case Western Reserve University 196211, Cleveland, Ohio, USA; 13 Center for Antimicrobial Resistance and Epidemiology, Louis Stokes Cleveland Veterans Medical Center, University Hospitals Cleveland Medical Center 114516, Cleveland, Ohio, USA; Houston Methodist Hospital and Weill Cornell Medical College, Houston, Texas, USA

**Keywords:** cystic fibrosis, lung transplantation, *Burkholderia cepacia *complex, multidrug resistance, cefiderocol, sulbactam–durlobactam

## Abstract

A 50-year-old woman with cystic fibrosis (CF) and lung transplantation developed persistent deep abscesses due to multidrug-resistant (MDR) *Burkholderia cenocepacia,* unresponsive to multiple antibiotics and surgeries. Combination therapy with sulbactam–durlobactam (SUL-DUR) and cefiderocol (FDC) along with surgical interventions led to clinical and radiographic stability. This report of a successful sulbactam–durlobactam and cefiderocol treatment highlights this targeted β-lactam-β-lactamase inhibitor combination as an option for refractory *B. cepacia* complex (BCC) infections.

## INTRODUCTION

*Burkholderia cepacia* complex (Bcc) is a significant pathogen in cystic fibrosis (CF), with intrinsic and acquired resistance to many antimicrobials (1). Biofilm persistence and association with lung function decline make Bcc infections particularly problematic after lung transplant, where immunosuppression increases the risk of invasive disease (2, 3). Pre-transplant colonization with *B. cenocepacia* is associated with higher post-transplant mortality (3, 4) and often precludes transplant eligibility. Resistance to standard agents underscores the need for novel therapies (5). Cefiderocol (FDC) and sulbactam–durlobactam (SUL-DUR) exhibit potent *in vitro* activity against select multidrug-resistant (MDR) gram-negatives such as members of Bcc and *Acinetobacter* spp. (6–8), though clinical data remain limited. This refractory MDR *B. cenocepacia* intraabdominal infection was successfully managed with a combination of FDC and SUL-DUR.

## CASE PRESENTATION

A 50-year-old woman with homozygous F508del cystic fibrosis (CF) underwent bilateral lung transplantation 14 years before presentation. Her pre-transplant course included gastroesophageal reflux managed by Nissen fundoplication and MDR *B. cenocepacia* colonization. Following alemtuzumab induction, she was maintained on tacrolimus, mycophenolate, and prednisone. Comorbidities included chronic kidney disease (creatinine clearance 27.5 mL/min), insulin-dependent diabetes mellitus (A1c 6.9%), and native tracheomalacia.

She had a history of multiple suspected antimicrobial-related adverse reactions and intolerances—rash with meropenem, rash and eosinophilic pleural effusion with ceftazidime, vancomycin infusion–related reaction with thrombocytopenia, cytopenias with linezolid, methemoglobinemia with dapsone, nausea with metronidazole, and rash with trimethoprim–sulfamethoxazole—necessitating cautious re-exposure and desensitization to antimicrobials.

Post-transplant, occasional episodes of *B. cenocepacia* tracheobronchitis were treated with multiple antibiotics including inhaled meropenem (MEM), ciprofloxacin, minocycline (MIN), as well as intravenous (IV) MEM and ceftazidime (CAZ) after desensitization. Trimethoprim-sulfamethoxazole (SXT) was avoided due to prior rash.

She presented to the hospital with low-grade fever, severe rectal pain, and leukocytosis. Imaging revealed right tubo-ovarian abscess with pelvic and perirectal inflammation and adhesions. Mycophenolate was discontinued. She received IV daptomycin and piperacillin–tazobactam [TZP] and underwent right salpingo-oophorectomy and lysis of adhesions. Intraoperative cultures grew *B. cepacia* complex (4+), later speciated by the Cystic Fibrosis Foundation *Burkholderia cepacia* Laboratory and Repository as *B. cenocepacia* (isolate #1). Antimicrobial susceptibility testing (AST) was performed using historical Clinical and Laboratory Standards Institute (CLSI) methods and interpretive criteria (9) (Table 1). She was transitioned to ceftazidime–avibactam [CZA] plus TZP, but after 3 weeks, she developed acute kidney injury and acute confusion with tremors, concerning for β-lactam–associated neurotoxicity in the setting of acute kidney injury, prompting discontinuation of therapy with resolution of symptoms in a few days. Antibiotics were discontinued after imaging showed findings suggestive of evolving postsurgical changes rather than ongoing infection.

**TABLE 1:** Timeline of cultures, isolate-level susceptibility data, and antimicrobial therapy decisions in relapsing MDR *Burkholderia cenocepacia* infection^*a*^

Timeline	Imagings/procedure	Antibiotic susceptibility testing	Antimicrobial treatment	Rationale
Initial presentation	Imaging : right tubo-ovarian abscess with pelvic and perirectal inflammation and adhesions Procedure: right salpingo-oophorectomy, lysis adhesions, and ureterolysis	Isolate #1 CAZ KB zone 0 mm [R] MEM KB zone 0 mm [R] SXT KB zone 0 mm [R] MIN KB zone 14 mm [R] I-R KB zone 14 mm FDC KB zone 30 mm CZA E-test MIC 8 µg/mL	Empirical: IV daptomycin and TZP Targeted: CZA + TZP Duration: ~3 weeks and stopped after developing neurotoxicity	CZA + TZP were used as a strategy to maximize β-lactam target engagement in a deep-seated, high-inoculum infection with limited therapeutic alternatives
~ 1.5 months from the initial presentation	Imaging: multiloculated fluid collection along the anterolateral aspect of segment V/VI of the liver with extension into the anterior abdominal wall musculature Procedure: Laparoscopic drainage of thick-walled abscess around the right upper quadrant Post-procedure positron emission tomography/computed tomography (PET/CT): See legend Fig. 1A	Isolate #2 CAZ KB zone 0 mm [R] MEM KB zone 0 mm [R] SXT KB zone 0 mm [R] MIN KB zone 18 mm [I] I-R KB zone 9 mm FDC KB zone 33 mm CZA E-test MIC 16 µg/mL	Empirical: CZA, MIN, and metronidazole Targeted: FDC and high-dose MIN Duration: FDC continued. MIN was eventually stopped within a month given arthralgias improved after discontinuation of MIN.	Recurrent right upper quadrant pain led to follow-up imaging demonstrating a multiloculated perihepatic collection that raised concern for persistent infection. After cultures confirmed MDR *B. cenocepacia* and antimicrobial testing demonstrated low FDC MICs as prior, therapy was transitioned to FDC with high-dose MIN. FDC was continued based on sustained *in vitro* activity and pharmacokinetic/pharmacodynamic (PK/PD) feasibility.
~4.5 months from the initial presentation	Imaging : See legend Fig. 1B Procedure: Right hepatic segment resection (segment VI), cholecystectomy, mobilization of the colon, and ureteral stent placement Post-procedure PET/CT: See legend Fig. 1C	Isolate #3 CAZ KB zone 0 mm [R] MEM KB zone 0 mm [R] SXT KB zone 0 mm [R] MIN KB zone 0 mm [R] I-R: KB zone 0 mm FDC KB zone 28 mm/MIC 0.25 µg/mL CZA E-test MIC 32 µg/mL SUL-DUR MIC 0.25 µg/mL	Empiric: SUL-DUR was added to FDC along with 7 days of MIN and levofloxacin for the peri-operative period. Targeted: FDC + SUL DUR Duration: continued for ~ 2.5 months post-procedure	PET/CT obtained to distinguish persistent infection from postoperative change when symptoms persisted; persistent FDG-avid, multifocal disease with relapse risk prompted empiric addition of SUL-DUR to FDC before MICs. Continued once low MICs supported activity. No SUL-DUR MIC was performed on prior isolates

^*a*^ CAZ, ceftazidime; MEM, meropenem; SXT: trimethoprim–sulfamethoxazole; MIN, minocycline; I-R, imipenem–relebactam; FDC, cefiderocol; KB, Kirby–Bauer); CZA, ceftazidime–avibactam; MIC, minimum inhibitory concentration (µg/mL); SUL-DUR, sulbactam–durlobactam. Resistant [R] and Intermediate [I] in brackets reflect historical CLSI Bcc disk criteria (context only); current breakpoints are unavailable/withdrawn, and MICs/zone diameters were interpreted supportively.

**Fig 1:**
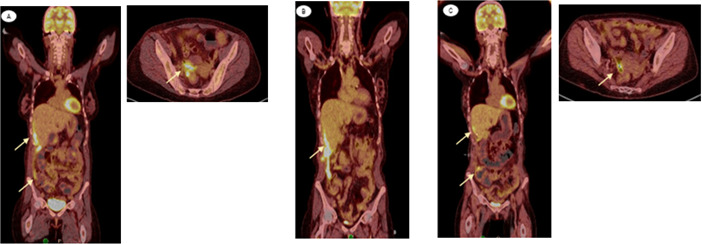
Trend in positron emission tomography/computed tomography (PET/CT) through course, with arrows showing areas of concern (**A**) Initial PET/CT obtained a month after laparoscopic abscess drainage showing heterogeneous, markedly avid fluorodeoxyglucose (FDG) uptake in the right perihepatic collection extending along the anterior abdominal wall musculature and right peritoneum (left), with additional focal uptake in the midline superior and right pelvis (right). (**B**) Repeat PET/CT 2 months after laparoscopic drainage, while on cefiderocol and minocycline, showing right liver margin uptake with a new contiguous collection extending into the right lower quadrant and focal thickening/fluid buildup. (**C**) PET/CT 1 month after right hepatic segment VI resection with cholecystectomy and mobilization of the colon from the abdominal wall showing fluid in the resection cavity measuring approximately 5.8 × 1.3 cm in the axial plane with FDG uptake surrounding the fluid collection and extending superiorly and anteriorly along the right hepatic capsule. Focus of FDG uptake in the right pelvis is decreased compared with prior imaging and was previously considered physiologic bowel activity, though residual inflammation and/or infection related to the prior tubo-ovarian abscess could not be excluded.

One month later, she was noted to have exertional right upper quadrant pain with a perihepatic collection presumed to be a resolving trocar-associated hematoma in the absence of other indicators of infection and eventually developed into a multiloculated fluid collection along the anterolateral aspect of segment V/VI of the liver with extension into the anterior abdominal wall musculature, concerning for Fitz–Hugh–Curtis syndrome. Empiric treatment with CZA, MIN, and metronidazole was initiated to provide broad aerobic and anaerobic coverage while she underwent laparoscopic drainage of this thick-walled abscess around the liver extending into the abdominal wall. Cultures grew MDR *B. cenocepacia* (isolate #2 with AST in Table 1). Postoperative positron emission tomography/computed tomography (PET/CT) showed fluorodeoxyglucose (FDG)-avid perihepatic and pelvic collections (Fig. 1A). She was discharged on FDC and high-dose MIN.

Despite antibiotics, PET/CT 2 months after laparoscopic drainage showed increased right liver margin FDG uptake, a new contiguous collection, and persistent pelvic inflammation (Fig. 1B). Vaginal swab demonstrated continued genital tract colonization with *B. cenocepacia*. MIN was discontinued due to arthralgias.

## CHALLENGE QUESTION

Which therapeutic strategy, along with surgical source control, best integrates susceptibility interpretation, likely resistance mechanisms, and pharmacological principles for this patient now?

- Continue cefiderocol (FDC) monotherapy; administer FDC as standard 30-min infusions since siderophore uptake overcomes resistance without PK/PD optimization.
- Initiate FDC plus sulbactam–durlobactam (SUL-DUR) recognizing that *Bcc* resistance involves multiple β-lactamases, efflux, and porin changes and that pairing FDC with a potent β-lactamase inhibitor regimen may enhance activity and suppress resistance.
- Restart ceftazidime–avibactam plus piperacillin–tazobactam given the prior partial response.
- Switch to high-dose meropenem by continuous infusion to overcome resistance through time-dependent exposure; avoid further surgery given prior drainage.
- Use colistin plus tigecycline to target persistent genital tract colonization and deep collections, accepting nephrotoxicity risk due to prior acute renal injury.

## TREATMENT AND OUTCOME

SUL-DUR was added to FDC. After 2 weeks of this regimen and an additional 7 days of levofloxacin and MIN along with FDC and SUL-DUR, she underwent hepatic segment V, VI resection, cholecystectomy, and adjacent peritoneum resection. SXT was also used briefly after desensitization but subsequently discontinued owing to transaminitis. Intraoperative cultures grew <1 + *B. cenocepacia* (isolate #3 with AST in Table 1). Elevated transaminases improved after SXT discontinuation. She was discharged on FDC and SUL-DUR with PET/CT 6 weeks postoperatively showing decreased pelvic FDG uptake and a stable perihepatic collection (Fig. 1C). Additional AST testing showed an FDC KB zone 28 mm and minimum inhibitory concentration (MIC) 0.25 µg/mL (via iron-depleted Mueller Hinton Broth per CLSI guidelines for FDC) (10, 11) and SUL-DUR MIC 0.25 µg/mL.

Two months postoperatively, she was readmitted with intractable nausea and vomiting, metallic mouth taste, and severe malnutrition but stable lab findings and imaging. FDC and SUL-DUR were held briefly and then resumed at reduced doses, but symptoms persisted. After a multidisciplinary discussion, all antimicrobials were discontinued. Total parenteral nutrition was initiated due to enteral intolerance and continued at home for several weeks. Serial imaging through 7 months following completion of the therapy demonstrated continued reduction in perihepatic inflammation.

## DISCUSSION

This case illustrates successful management of intra-abdominal MDR *Burkholderia cenocepacia* infection in a lung transplant recipient—an uncommon extrapulmonary manifestation. Although *B. cenocepacia* typically colonizes the respiratory tract in CF, its biofilm-forming capacity impairs neutrophil function and innate immunity, enabling dissemination under immunosuppression (12). Vaginal Bcc colonization despite therapy suggests that perhaps the genital tract was colonized via the gastrointestinal tract following the swallowing of pulmonary secretions. The organism can extend directly from the pelvis along the paracolic gutters or via the lymphatics to reach the peritoneum surrounding the liver, leading to Fitz–Hugh–Curtis syndrome (13). Because routine clinical methods may misidentify species within the Bcc, confirmation at a reference laboratory may be important when species-level distinctions influence prognosis, infection-control considerations, or therapeutic decision-making.

Treatment success reflected a coordinated, multidisciplinary strategy. Bcc’s resistance mechanisms—including efflux pumps, inducible Ambler class A, C, and D β-lactamases (e.g*.,* PenA and AmpC), porin loss, and penicillin-binding protein (PBP) modifications—limit monotherapy efficacy and predispose to relapsing infection despite surgical source control.¹˒² FDC and SUL-DUR were selected based on emergence evidence of activity against carbapenem-resistant gram-negative pathogens, including Bcc and *Acinetobacter baumannii* (6–8, 14–16).

**Fig 2:**
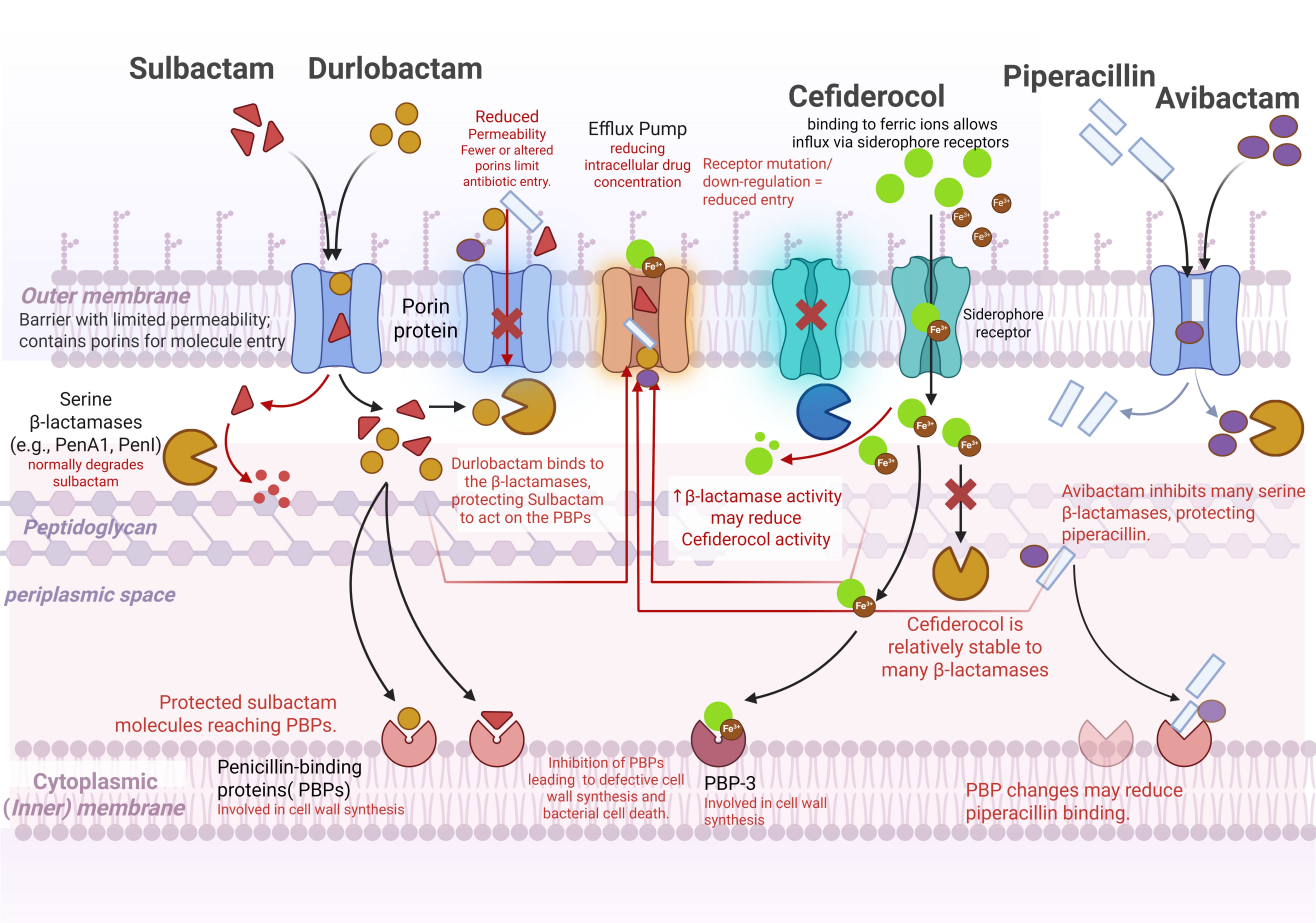
Illustrated mechanisms of action and resistance for sulbactam–durlobactam, cefiderocol, and piperacillin–avibactam in the cross‐section of a *Burkholderia cepacia* complex cell envelope. Sulbactam (red wedges) enters the periplasm via porins and inhibits PBPs (predominantly PBP1a>2 > 3) involved in peptidoglycan synthesis; intrinsic β-lactamases (e.g., PenA/PenI) can hydrolyze sulbactam. Durlobactam (yellow circles), a diazabicyclooctane β-lactamase inhibitor, inhibits serine β-lactamases and protects sulbactam, allowing sufficient active drug to reach PBPs and disrupt peptidoglycan synthesis, resulting in bacterial cell death. Reduced permeability (porin loss/alteration) and increased efflux may decrease sulbactam–durlobactam concentration and contribute to resistance. Cefiderocol (green circles) chelates Fe³^+^ and uses TonB-dependent siderophore receptors for uptake and then binds PBP3 to inhibit cell wall synthesis. Cefiderocol nonsusceptibility may result from impaired siderophore receptor/iron-transport pathways, efflux, and/or increased additional β-lactamase activity. Piperacillin (blue rectangles) enters the periplasm via porins and targets PBPs to inhibit peptidoglycan synthesis. Avibactam (purple ovals), a diazabicyclooctane β-lactamase inhibitor, inhibits many serine β-lactamases (class A/C and some D), thereby protecting piperacillin from hydrolysis. Reduced permeability, Resistance-Nodulation-Division (RND) efflux, β-lactamase induction/overexpression, and PBP alterations may limit activity and contribute to on-therapy resistance. Icon key: yellow pac-man enzyme = serine β-lactamases (classes A/C/D); blue enzyme = metallo-β-lactamases; blue/teal channels = porins/siderophore receptors (permeability and “Trojan-horse” entry); orange pump = RND efflux pumps; pink pac-man proteins = PBPs. Faded pink pac-man proteins: altered PBPs. Figure generated using BioRender.

FDC uses bacterial iron uptake pathways for periplasmic entry and resists hydrolysis by diverse β-lactamases.⁶⁻⁸ SUL-DUR restores sulbactam’s bactericidal action through durlobactam-mediated inhibition of Ambler class A, C, and D β-lactamases (15, 16). DUR’s low apparent inhibition constant (Ki_app 15-241 nM) against class D enzymes preserves SUL function and limits resistance (17) (illustrated in Fig. 2). Although durlobactam has been shown to bind PBPs, including PBP2, in other gram-negative organisms, whether similar PBP interactions occur in *B. cenocepacia* remains unknown as no structural or homology studies have characterized PBP binding in this species (7, 17). Murine pharmacokinetic/pharmacodynamic (PK/PD) studies established SUL free drug concentration over time (fT)>MIC targets (~20%–45%) and DUR thresholds (≥0.75 µg/mL) for bacterial stasis and ≥1 log₁₀ CFU reduction, guiding human dosing (7, 18).

In the PERSEUS subgroup of difficult-to-treat gram-negative infections—including Bcc—FDC achieved clinical cure rates of 62.5%–80%, supporting its utility (19). Furthermore, time-kill assays show that FDC combined with β-lactamase inhibitors—particularly SUL—achieves >3 log₁₀ CFU reduction at sub-MIC levels and suppresses resistance emergence (20). SUL-DUR received FDA-accelerated approval in May 2023 for *A. baumannii* infections (21). In the ATTACK phase 3 trial where SUL-DUR and colistin were administered in combination with background imipenem–cilastatin, SUL-DUR achieved noninferiority to colistin (28-day mortality 19% vs 32%) and significantly less nephrotoxicity (13% vs 38%; *P* < 0.001) (22). Early post-marketing and real-world reports in critically ill and transplant cohorts support its favorable safety profile, with only mild gastrointestinal upset and transient hepatic enzyme elevations (23).

Our patient’s care was complex due to MDR *B. cenocepacia* with limited susceptibility, multiple drug allergies requiring desensitization, and concern for cumulative toxicities. Antimicrobial selection in this case was guided by microbiologic, pharmacodynamic, and mechanistic considerations. Early use of CZA plus TZP reflected limited options for MDR Bcc and an attempt to maximize β-lactam target engagement in a deep-seated, high-inoculum infection, despite limited supporting clinical data. The elevated CZA MIC and extensive prior β-lactam exposure also raised concern for inducible β-lactamase expression (e.g., PenA), underscoring the risk of on-therapy resistance emergence and supporting transition to alternative agents with distinct entry and inhibition mechanisms. Transition to FDC and SUL-DUR was supported by low isolate MICs, its complementary mechanisms of action, and by the known multifactorial resistance phenotype of Bcc. While either agent could be considered monotherapy, combination therapy was favored given the relapsing course, prolonged treatment duration, and concern for resistance emergence in the absence of isolate-specific molecular resistance data.

She tolerated dual therapy with FDC and SUL-DUR for 2.5 months alongside surgical intervention, achieving sustained disease control with no recurrence as of 7 months following completion of treatment. Immunosuppression was cautiously attenuated to support host defense while preserving graft function. Gastrointestinal intolerance was likely multifactorial, driven by repeated abdominal interventions, ongoing inflammatory burden with malnutrition, and prolonged antimicrobial exposure, and improved following antimicrobial discontinuation and parenteral nutritional support.

No consensus exists regarding the interpretation of susceptibility testing for Bcc. CLSI withdrew breakpoints for agents like FDC and CZA against Bcc (2024 M100 update) (10, 11), citing insufficient PK/PD and clinical data (24). The European Committee on Antimicrobial Susceptibility Testing likewise does not provide breakpoints for these agents, underscoring a global unmet need and the importance of an international collaboration. Accordingly, AST results in this case were interpreted as supportive measurements rather than definitive susceptible/resistant categories, integrating MICs and disk diffusion zone diameters with PK/PD feasibility and clinical context. Cefiderocol testing was performed using CLSI-recommended methodology (iron-depleted media), and the observed low MIC/zone diameters for cefiderocol and sulbactam–durlobactam supported achievable β-lactam exposures when paired with aggressive source control and serial imaging follow-up. Formal *in vitro* synergy testing (e.g., checkerboard or time-kill assays) was not performed on the clinical isolates; therapeutic decisions were based on isolate-specific susceptibility data, infection complexity, and available *in vitro* and clinical literature rather than as sole determinants of therapy. Strain-relatedness testing and whole-genome sequencing were not performed but would be valuable to confirm clonality and characterize within-host resistance evolution (including rising ceftazidime–avibactam MICs) and virulence determinants in relapsing Bcc infection.

Serial PET/CT imaging provided functional correlation with clinical symptoms and guided therapeutic escalation by distinguishing persistent metabolically active infection from postoperative change, particularly when conventional imaging and laboratory markers were equivocal (25). Although definitive hepatic/peritoneal resection likely provided the key source control, serial PET/CT demonstrated multifocal FDG-avid disease (including persistent pelvic activity), supporting FDC plus SUL-DUR as adjunctive therapy for residual infection/inflammation beyond a single drainable focus.

In summary, FDC plus SUL-DUR led to sustained clinical and radiological stability in our lung transplant recipient with MDR Bcc, facilitated by a multidisciplinary approach involving these novel antimicrobials, surgical source control, and carefully attenuated immunosuppression. Prospective studies should evaluate its optimal dosing, long-term safety, and integration into regimens for high-risk populations such as lung transplant recipients.
